# Online and Offline Intervention for the Prevention of Postpartum Depression among Rural-to-Urban Floating Women: Study Protocol for a Randomized Control Trial

**DOI:** 10.3390/ijerph19137951

**Published:** 2022-06-29

**Authors:** Xichenhui Qiu, Ting Li, Qiyu Fang, Lingling Huang, Xujuan Zheng

**Affiliations:** 1 School of Nursing, Health Science Centre, Shenzhen University, Shenzhen 518060, China; qiuxichenhui@szu.edu.cn (X.Q.); fangqiyu320@szu.edu.cn (Q.F.); huanglingling@szu.edu.cn (L.H.); 2 Department of Obstetrics, South China Hospital, Health Science Center, Shenzhen University, Shenzhen 518116, China; 15876509280@163.com

**Keywords:** postpartum depression, online and offline intervention, postpartum women, e-health technology, social stigma, social support, study protocol

## Abstract

Background: As a higher-risk group of postpartum depression (PPD), rural to urban floating women urgently require effective and accessible mental health care after childbirth to prevent PPD. Even though there were various interventions, only a small number of women have sought professional help to reduce their depressive symptoms after childbirth, suggesting the need for an innovative intervention delivery to overcome women’s help-seeking barriers. Online and offline (OTO) interventions, which combine face-to-face and internet-based interventions, provide apparent benefits. As a result, the protocol for a randomized controlled study (RCT) was designed to examine the effectiveness and acceptability of OTO intervention on psychosocial outcomes for Chinese rural-to-urban floating women including the reduction of PPD symptoms and PPD stigma, and the improvement of social support and quality of life. Methods: A double blind, multicenter, RCT will be used and a total of 226 participants will be recruited. The OTO intervention called the “Hi, Mom” program will integrate two face-to-face consulting sessions with online sessions comprising an information module, a communication module, an ask-the-expert module, and a peer story module over a period of three months. The control group will receive routine postpartum care. Outcome measures including PPD symptoms, PPD stigma, social support, quality of life, mother–child bonding, and satisfaction with health care received will be conducted at baseline, postintervention, and three-month follow-up. Results and Discussion: If the intervention is effective, it will provide a convenient and effective intervention program on postpartum mental well-being for rural-to-urban floating women. As the first study to test the effects of an OTO intervention for the prevention of PPD in China, the outcomes gained from this study will provide evidence-based knowledge for clinical practice on PPD prevention based on online and offline health technologies. Moreover, it could be used to plan a culturally appropriate OTO intervention for migrant mothers from different countries.

## 1. Introduction

Postpartum depression (PPD) is defined as any major or subclinical depression occurring in women after childbirth [1]. In the recent ten years, PPD has become one of the severe worldwide public health problems [2,3], and its incidence rate varies from about 20% in developed countries to 25% in developing countries [4]. In addition to the high prevalence, PPD was identified to have adverse effects on the physical and mental health of women [3], the cognitive and emotional development of babies [5], and the bonding of family members [6]. In consideration of the negative consequence of PPD, it is of significance to carry out efficient and timely interventions [7].

Systematic review and meta-analysis found from [7,8,9,10] that there have been various interventions for PPD, i.e., pharmacological interventions and nonpharmacological intervention. Although pharmacological method is an effective treatment for depression, mothers are often reluctant to take antidepressant medication due to concerns about breast milk transmission or potential side-effects on their babies [8,11]. Thus, it is important to evaluate the effects of nonpharmacologic interventions on postpartum women experiencing PPD. In the existing literature, psychosocial intervention [11,12,13,14,15] and physical intervention [16,17,18,19] have been identified to be associated with a reduction in the likelihood of continued PPD, which mainly used traditional face-to-face method.

Despite the existence of these traditional interventions, there have been few women with PPD seeking professional help [7,20]. For instance, a cohort study including 1126 women in Hunan, China, found that only 9.3% of women at a high risk of PPD chose professionals as their first option [21]. Another study revealed that only 13.6% of Portuguese women with PPD sought professional help to address their emotional difficulties during the postpartum period [22]. Actually, some factors hindered the feasibility and generalizability of these traditional interventions. Firstly, the shortage of health professionals negatively affected the accessibility of traditional mental health services [23,24]. Secondly, the stigma on PPD blocked women seeking face-to-face help, which was particularly prominent in developing countries [25]. Thirdly, time and financial constraints and struggles with parenting babies were the other obstacles of women in attending traditional face-to-face intervention [24]. These barriers indicated the urgent need for a new form of intervention to improve the access of postpartum women.

E-health technology was the innovative approach of intervention delivery that uses online technology [26], with the distinguished advantages of improved flexibility, accessibility, and reduced financial and time cost [27]. The random-effects model of a recent meta-analysis indicated that the internet-based interventions have proven to be effective in the reduction of PPD symptoms (d = 0.642, N = 7) [13]. However, all the seven randomized controlled trials (RCTs) for PPD based on internet had been conducted in developed countries, such as UK, USA, Canada, Australia, and Sweden [13]. Furthermore, some researchers argued that internet-based interventions had obvious drawbacks of low diagnosis accuracy, and engagement and response rates due to the absence of face-to-face support and assessment from health professionals during the whole intervention [28,29,30]. Moreover, internet-based intervention lacked important opportunities to provide customization for ensuring relevance [31]. Thus, online intervention can be a significant supplement of traditional interventions, rather than replacing them [32].

Online and offline (OTO) intervention combines face-to-face (meaning directly meeting someone in the same place and allowing for the full potential of nonverbal communication) therapy with internet-based interventions, which can be integrated and sequentially conducted. Previous research verified that the blended OTO intervention had positive outcomes on some psychological disorders, such as depression [31,33,34], and was highly accepted by patients [35]. To the best of our knowledge, no RCTs by online and offline intervention have been conducted targeting PPD in China.

During the last three decades, there have been a great number of people moving from rural areas to urban areas owing to the rapid development of the economy and urbanization in China [36,37]. These special people, called “floating population” or “internal migrants”, are approximately 247 million, accounting for almost one-fifth of the total population in China [38]. Reports said almost half of floating populations were women, and the majority of them were in childbearing age [39,40]. In comparison with city residents, these floating women had characteristics of a younger childbearing age, having lower educational levels, and poor living and working environments [39,40].

Evidence indicated that health inequality has obviously existed between the city residents and the floating women due to the imbalance of health resources allocation [41,42]. Prior studies found that floating women were prone to encountering more mental problems and receiving less social support during the postpartum period in comparison with city residents [43,44,45]. For example, the mean EPDS (Edinburgh Postnatal Depression Scale) scores among rural-to-urban floating women at 6 and 12 weeks postpartum were 11.19 (4.89) and 11.18 (5.34), respectively [45]; much higher than the corresponding results of 9.09 (4.33) and 8.63 (4.41) from city resident women [46]. International research had similar findings that the incidence of PPD in American mothers who are of nondominant culture and socioeconomically disadvantaged had three to four times higher scores than in women from the general population [47].

As a higher-risk group of PPD, rural-to-urban floating women urgently require effective and accessible mental health care after childbirth; and the future tailored, evidence-based intervention is highly needed to improve these women’s psychological well-being and social support during the postpartum period [44,45]. Therefore, the protocol for a randomized controlled trial was designed in the research to examine the acceptability and effectiveness of an online and offline (OTO) intervention for PPD prevention among rural-to-urban floating women.

## 2. Methods

### 2.1. Aim

The purpose of this study was to examine the effectiveness of OTO intervention on psychosocial outcomes for Chinese rural-to-urban floating women, including the reduction of depression symptoms and PPD stigma, and the improvement of social support, quality of life, and satisfaction.

We hypothesized that compared with floating women in the control group; participants in the intervention group will be reported to have the significant differences:

(1) Reduced PPD symptoms and stigma;
(2) Improved social support, quality of life, and mother–child bonding;
(3) Great satisfaction with health care received during the postpartum period.

### 2.2. Design

A double blind, multicenter, randomized controlled trial (RCT) was designed in the research. The SPIRIT 2013 Statement and the guidelines for the Standard Protocol of Clinical Trials are strictly followed.

### 2.3. Participants

Participants who meet the inclusion criteria will be recruited. The inclusion criteria are: (1) rural-to-urban floating women; (2) ≥18 years old; (3) married; (4) women with an EPDS (Edinburgh Postnatal Depression Scale score) of 10 or more; (5) women with access to the internet. The exclusion criteria are: (1) having depression history; (2) with a serious physical or mental condition of either the mother or the infant; (3) undergoing psychotherapy or any other physical or psychological intervention.

### 2.4. Study Setting and Recruitment

**Figure 1:**
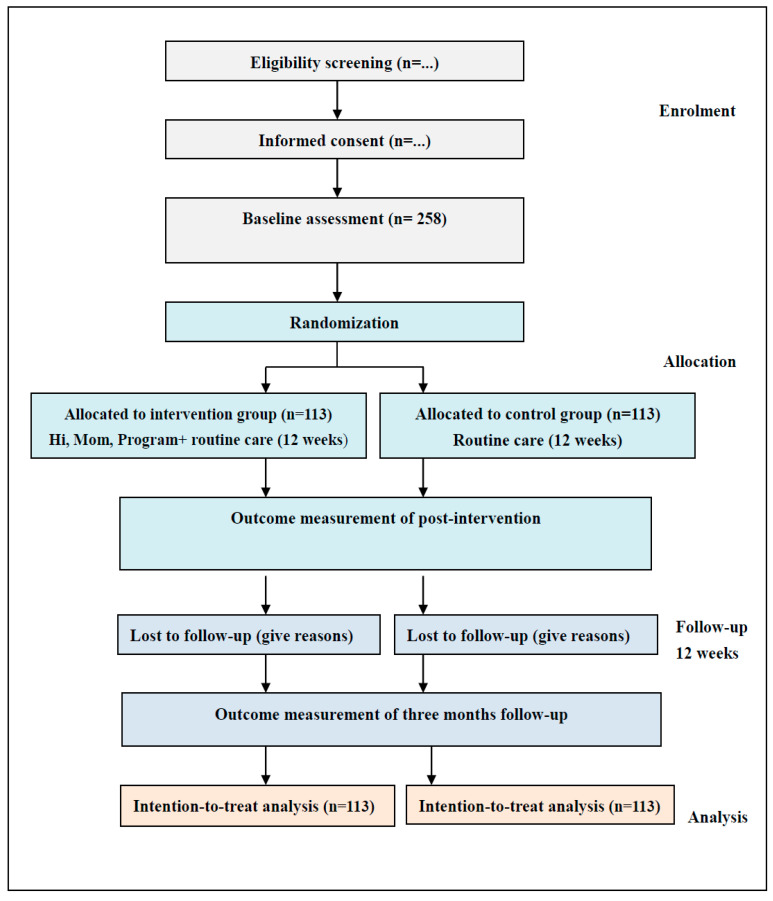
CONSORT flowchart of the study.

The research will be conducted in the maternity wards of three affiliated hospitals in X University, where each hospital has an annual baby-birth record of more than 4000. All women admitted in the wards will be approached by researchers to ask their willingness and will be measured by the EPDS before recruitment. If they have an EPDS of 10 or more, they will be eligible to participate in this study. The information sheets, posters, and leaflets will be used to introduce the purposes and process of the research before recruitment. If rural-to-urban floating women meeting the inclusion criteria are willing to participate, they will be asked to sign an informed consent form and be informed of freedom to withdraw whenever they want. The CONSORT (consolidated standards of reporting trials) [48] flowchart is illustrated in Figure 1. The recruitment period is expected to extend over a 3-month period. Recruitment of participants began in May 2022. Primary endpoints (baseline and postintervention), follow-up measure (three months), and data analysis are expected to be complete in January 2023.

### 2.5. Sample Size

The study sample size will be determined by the primary outcome of PPD, with the standardized effect size of 0.35 for PPD (EPDS scores) reported in a previous PPD trial [49,50]. A sample of 180 women (90 women per group) will be needed to detect an effect size of at least 0.35, with 80% power, two-sided *p* < 0.05, which is calculated by inputting the above parameters in PASS (Power Analysis and Sample Size) software (NCSS company, East Kaysville, UT, USA). Assuming an attrition rate of about 20%, a minimum of 226 women (113 in each group) is required.

### 2.6. Randomization and Blinding

Participants will be randomly assigned to the intervention group or control group with an allocation ratio as 1:1. Sequence randomization will be performed using a computerized random number generator, and its result will be kept in sealed and opaque envelopes and saved by the researchers who have no direct relationship with the project to make sure that the random allocation is masked in advance. During the research process, the group allocation will be blinded to the participants, enrolling researchers, outcome assessors, and data analysts.

### 2.7. Intervention

**Figure 2:**
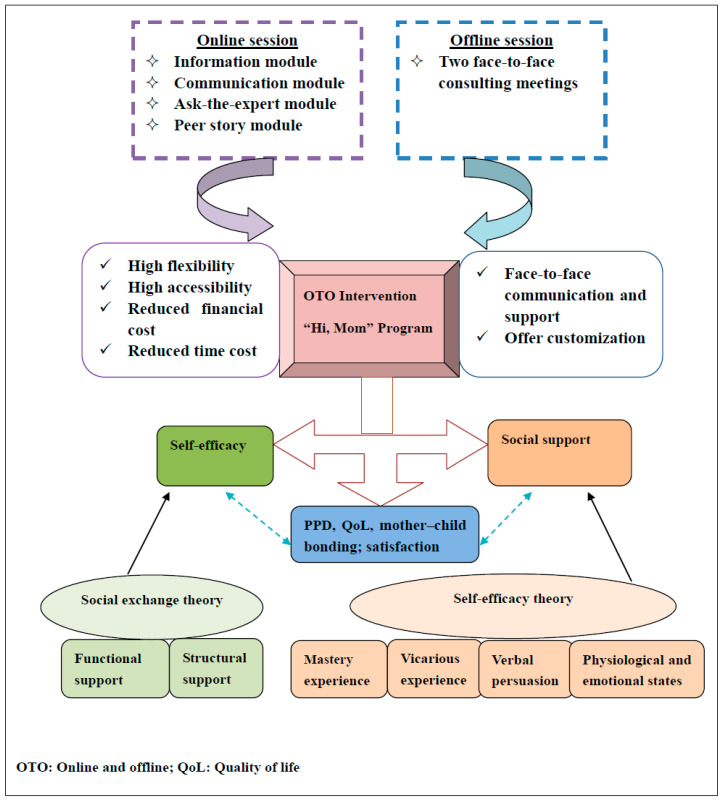
The theoretical framework of the “Hi, Mom” intervention.

An online and offline (OTO) intervention for PPD prevention among rural-to-urban floating women, called the “Hi, Mom” program, was developed using the theoretical framework incorporated self-efficacy theory [51] and social exchange theory [52]. Sound evidence identified that appropriate levels of self-efficacy and social support are essential components of interventions to improve patients’ symptom management [23]. The “Hi, Mom” program is designed to enhance women’s self-efficacy and social support to promote their ability to manage their depression symptoms, then improve their psychological well-being and quality of life. The theoretical framework of the “Hi, Mom” is shown in Figure 2.

Self-efficacy, defined as the degree of one’s feelings about his/her ability to accomplish goals, was affected by four significant factors of mastery experience, vicarious experience, verbal persuasion, and physiological and emotional states [51]. Mastery experiences can be accumulated through past experiences. Vicarious experiences are obtained from the observation of others (i.e., role model) to achieve success in similar situations. Verbal persuasions are correlated with other person’s comments, feedback, and suggestion. Physiological and emotional states mean people’s physical and mental status. Social support is defined as the perception of possible received supports and the satisfaction with accepted assistances, comprising the structural and functional components [52]. Of which, the structural social support includes a formal social network (i.e., from health professionals) and informal social network (i.e., from family members, friends); while the functional social support is divided into informational support, material support, emotional support, and evaluation of support [53].

The “Hi, Mom” program of OTO intervention will integrate two face-to-face consulting sessions with online sessions including an information module, a communication module, an ask-the-expert module, and a peer story module over a period of three months. According to the self-efficacy theory, the mastery experiences of participants will be acquired by provision of the related PPD information via the information module, ask-the-expert module, and face-to-face consulting. Vicarious experiences of women can come from the peer support module where women may share their successful experience of dealing with PPD. Verbal persuasion can be gained from feedback, comments, and suggestions from health professionals in the ask-the-expert module and other mothers in the communication module. The physiological and emotional states can be improved through the above various supports, information and feedback in the information module, communication module, ask-the-expert module, peer story module, and face-to-face consulting. In terms of the social exchange theory, the structural social support, including the formal and informal social network of women, can be built; and various kinds of functional supports, such as informational support, material support, emotional support, and evaluation of support, can be provided from the health professionals and others through the designed information module, communication module, ask-the-expert module, peer story module, and face-to-face consulting.

The women in the control group will receive routine care, including health care from obstetricians and obstetric nurses during the hospitalization of three to five days; and at least two home visits from community health professionals [54]. The women in the intervention group will receive an OTO intervention called the “Hi, Mom” program and routine postpartum care. Table 1 describes the details of the “Hi, Mom” program.

The online session of the “Hi, Mom” program is designed to have fixed structures, which lasts approximately three months. Four modules are incorporated to meet learning needs, communication needs, emotional needs, and evaluation needs for rural-to-urban floating women. The information module is the PPD learning forum about prevention knowledge and skills showed by multimedia resources. In consideration of the lower educational level of floating women, we prefer to use many more pictures and videos, rather than words. The related knowledge and skills include (1) How to understand PPD? (2) How to deal with negative emotions? (3) How to relax yourself? (4) How to combat the stigma? (5) When and how to seek help? (6) How to balance postpartum life and family relationship? (7) How to adapt to the role of a mom? 

The communication module, ask-the-expert module, and peer story module will provide women with opportunities for acquiring social networking and various kinds of supports. For example, the communication module provides a platform for new mothers to tell their own postpartum stories, exchange experiences with PPD, and seek empathy and belonging. This module also invites women to discuss some topics such as the experience of “Doing the month”, communication barriers, potential interpersonal conflicts after childbirth, and effective skills to solve emotional problems. The ask-the-expert module gives floating women opportunities of timely interaction with health professionals where experts can answer women’s questions within 24 h. Some self-testing tools are likewise included in the module to assist new mothers in self-assessing their state of mind. The peer story module is the last module where six mothers are invited to share their successful experience of overcoming PPD and provide role models for these floating women. 

Floating women in the intervention group will be taught to log in and use each online module of the “Hi Mom” program. Women will be required to log into the online sessions at least twice a week, totaling at least one hour per week; and their duration and frequency of logins will be monitored to evaluate an individual’s adherence. Technical assistance for the online program will be available from Monday to Friday by telephone or email.

The offline session of the “Hi, Mom” program asks women to attend two face-to-face consulting meetings when the researchers conduct home visiting. Each face-to-face session will last an approximate length of 45–60 min, in which the researchers begin with mood checking and discussion of the women’s symptoms, then answer their questions, and provide face-to-face personalized, customized supports according to the various needs of each woman. 

Participants will receive phone reminders every week to motivate them to participate in the online and offline sessions. Risk assessment through both self-report of the women and the specific suicidal intention item on the EPDS questionnaire will be conducted during the intervention, postintervention, and at follow-up assessment. If the woman has a high risk of suicide, or a high likelihood of harming others, or develops severe depressive symptoms, she will be immediately referred to psychological or psychiatric health services, and her participation in the “Hi, Mom” program will end.

### 2.8. Measures

Table 2 presents the study variables and assessment time points.

#### 2.8.1. Social-Demographic and Clinical Data

Social-demographic and clinical data including maternal age, marital status, educational level, occupation, average monthly income, mode of childbirth, number of children, infant gender, infant feeding pattern, infant health, and infant fussiness will be collected through a questionnaire developed by the researchers.

#### 2.8.2. Primary Outcome

Postpartum depression symptoms as the primary outcome will be measured with the Chinese version of the Edinburgh Postnatal Depression Scale (EPDS). EPDS is composed of ten items and each item scores from 0 to 3 [55]. The total score ranges from 0 to 30, and the higher score indicates the more serious depressive symptoms. The reported internal consistency of the Chinese version EPDS was 0.87, and its concurrent validity was 0.79 with the BDI (Beck Depression Inventory) [56]. The total EPDS score of 10 or more is recommended as the presence of clinically relevant depressive symptoms in mainland China [56].

#### 2.8.3. Secondary Outcomes

Women perceived mental health-related stigma will be assessed by the Stigma-9 Questionnaire (STIG-9) [57]. The STIG-9 consists of nine items assessing cognitive, behavioral, and affective aspects of perceived mental health-related stigma. A four-point Likert scale will be used, i.e., disagree (0), somewhat disagree (1), somewhat agree (2), agree (3). Higher scores indicate stronger expectations of negative beliefs, feelings, and behaviors towards mentally ill people. The scale was found to have high internal consistency [57].

Postnatal social support scale (PSSS) will be used to assess Chinese women’s perception of received support in the postpartum period [58]. The 20-item tool is based on a four Likert-type point, being scored from 0 (never) to 3 (often). The total score of the PSSS is about 0–60. The higher score the mother has, implicates the more social support she receives. The reported internal consistency of this tool was 0.89 [58]. The test–retest reliability coefficient of PSSS was 0.92, and its content validity was 0.90.

Quality of life will be assessed with the Chinese version of the Euroqol Five-Dimension Scale (EQ-5D) [59]. It comprises five health dimensions (mobility, self-care, usual activities, pain/discomfort, and anxiety/depression), each with three response categories (no, some, or extreme problems) and a 0–100 points visual analogue scale (EQ VAS). The total score describes the health state, which is obtained through a fixed algorithm. The Chinese version of EQ-5D demonstrated an acceptable construct validity and test–retest reliability [59].

Mother–child bonding will be measured with the Chinese version of the Postpartum Bonding Questionnaire (PBQ) [60], which was developed to assess mother–infant bonding disturbances in the postpartum period. The 12-item instrument uses a six-point Likert scale ranging between 0 (never) and 5 (always). A lower score is indicative of a less impaired mother–child bond. The Chinese version of PBQ is found with good levels of internal consistency and validity and has been identified as a useful instrument for detecting mother–infant relationship impairments as perceived by Chinese women with PPD [60].

Women’s satisfaction with health care in the postpartum period will be assessed with a three-item questionnaire developed by the researchers. The items are “Overall, how satisfied with the healthcare your received?”, “Please give your reasons”, and “Please give your advice”.

### 2.9. Data Collection

Baseline assessments will be conducted by the research team and participants will be asked to complete EPDS, STIG-9, PSSS, EQ-5D, PBQ, and sociodemographic and clinical data in the obstetric wards. Postintervention assessment will be performed immediately after intervention and follow-up assessment will be performed 3 months after intervention. At the two time points of postintervention and follow-up assessment, the questionnaires, comprising EPDS, STIG-9, PSSS, EQ-5D, PBQ, and the health care satisfaction questionnaire, will be sent to participants by WeChat or email, and the completed questionnaires will be returned to the researchers likewise by WeChat or email. In order to improve the response rate, a reminder telephone or WeChat will be given to participants before and after one week of the two time points, respectively.

### 2.10. Data Analysis

All the statistical analyses will be conducted by the Statistical Package for Social Sciences (SPSS). Descriptive analysis will be undertaken to describe the social-demographic and clinical data. Mean, standard deviation, and frequencies, as well as percentages will be used for continuous data and categorical data, respectively. The chi-square (χ^2^) for categorical variables, and the independent sample *t*-test for continuous variables will be conducted to detect any significant difference on social-demographic and clinical characteristics and baseline outcomes between the intervention group and the control group. The effect of intervention on reduced PPD symptoms and stigma and on improved social support, quality of life, and mother–child bonding across the three time points will be evaluated via repeated measures multivariate analysis of covariance to explore how outcomes have changed between groups, over time, and the interaction between group and time. The independent *t*-test will be used to compare satisfaction with the health care that they received in the postpartum period between the intervention group and the control group on the time point of immediately after the intervention. A *p* value less than 0.05 will be considered statistically significant.

### 2.11. Ethical Consideration

The plan and the design content have been reviewed and approved by the Institutional Review Board of the University. This study will adhere to the ethical standards of the Declaration of Helsinki for the whole procedure. Information of all participants will be confidential and anonymized and only be treated at a collective level.

### 2.12. Validity and Reliability

The scientific and rigorous research design, i.e., the sound theoretical framework of intervention, a good representative and predetermined sample, and all the tools with a proven good reliability and validity, will be strictly executed to reduce bias effectively and enhance the generalizability of the research results beyond the target population. Furthermore, to ensure proper administration of the interventions, the related researchers will be trained before data collection. Moreover, the group allocation will be blind to the participants, enrolling researchers, outcome assessors, and data analysts in order to reduce the bias during the research process.

## 3. Discussion

Nowadays, the high prevalence of PPD for rural-to-urban floating women [44,45] and the shortage of mental health service resources in China [23] make it extremely urgent to effectively intervene in PPD for floating women. Despite the existence of various interventions, only a small number of women have sought professional help to reduce their depressive symptoms after childbirth [21], suggesting the need for an innovative intervention delivery to overcome women’s help-seeking barriers. Online and offline (OTO) intervention is the combination of face-to-face treatment with internet-based interventions that have significant benefits [7]. On the one hand, it can have higher flexibility and accessibility, and reduced financial and time cost [27]. On the other hand, it likewise can offer significant customization via face-to-face communication and support [7]. Therefore, our research aims to examine the effectiveness and acceptability of an OTO intervention of the “Hi, Mom” program on psychosocial outcomes for Chinese rural-to-urban floating women, including the reduction of PPD symptoms and PPD stigma, and the improvement of social support and quality of life. To the best of our knowledge, no RCTs by OTO intervention have been conducted targeting PPD in China.

Despite the advantages of the research, some limitations need to be noted. Firstly, self-report tools will be used to measure PPD, and it may lead to social desirability bias owing to the traditional belief of “domestic shame should not be made public”. Secondly, this study will not conduct further follow-up after three months’ intervention due to the time and financial restrictions. Thirdly, some potential biases could be caused as blinding of researchers during the whole research process is not possible.

## 4. Conclusions

It will be the first study to test the effects of an OTO intervention for the prevention of PPD among rural-to-urban Chinese floating women. This innovative format of intervention could possibly reduce financial and time costs in health care systems, increase the efficacy and outcome of mental health services, and promote help-seeking behaviors of Chinese floating women after childbirth. If the “Hi, Mom” intervention is verified to be effective in the prevention of PPD, this will not only provide a convenient intervention program for postpartum mental well-being among rural-to-urban floating women, but also will provide evidence-based knowledge for PPD clinical practice based on online and offline health technologies to save mental health service resources. Moreover, the outcomes gained from this study could be used to plan a culturally appropriate OTO intervention for other groups of postpartum women, such as migrant mothers from different countries, single mothers, and city resident women.

## Figures and Tables

**Table 1:** The details of the “Hi, Mom” program.

Items	Intervention Group	Control Group
Brief name	“Hi, Mom” + routine care	Routine care
Who provided	Researchers	Obstetricians, obstetric nurses, community doctors
Where	Web environment, home	Hospital, home
How (medium)	Online intervention by internet Offline intervention by face-to-face	Routine care by face-to-face
What	Information module; communication module; ask-the-expert module; peer story module; and face-to-face consulting.	Routine postpartum care, home visiting.
How long and how often	From childbirth to 3 month postpartum. The total intervention time not less than 12 weeks. Reminder telephones every week, reminding them to log in the online session at least twice a week, and no less than total 1 h per week; and attending 2 face-to-face consulting meetings, about 30–45min each time.	From childbirth to 1 month postpartum. At least 2 home visits.
When assessed	Baseline (preintervention) Postintervention (immediately after intervention) Follow-up (three months after intervention)	Baseline (preintervention) Postintervention (immediately after intervention) Follow-up (three months after intervention)

**Table 2:** The study variables and assessment times.

Variables	Baseline (T0)	Postintervention (T2)	Three Months Follow-Up (T3)
Sociodemographic and clinical data	※		
Postpartum depression symptoms	※	※	※
Postpartum depression stigma	※	※	※
Social support	※	※	※
Quality of life	※	※	※
Mother–child bonding	※	※	※
Satisfaction with health care received		※	

T0: before randomization; T1: immediately after the intervention; T3: three months after the intervention.

## Data Availability

Not applicable.
